# Editorial

**Published:** 1844-11-30

**Authors:** 


### THE MEDICAL EXAMINER.

PHILADELPHIA, NOV. 30, 1844.

### INTRODUCTORY LECTURES.

The winter courses of lectures having commenced in the various Medical Colleges of the country, we may expect shortly to be supplied with an abundance of these interesting annuals. We shall endeavour, from those sent to us, to cull many a literary flower, and many wholesome truths, "fitly expressed," for the amusement and instruction of our readers.

The "first fruit of the season" is now before us. It is "On the formation of Professional Character: an Introductory Lecture, delivered Nov. 4, 1844, by John P. Harrison, M. D., Professor of Materia Medica and Therapeutics in the Medical College of Ohio." In this lecture, the Professor treats of the dignity and importance of professional character to a physician, and the proper mode of acquiring it.

The doctrines inculcated are sound, the sentiments expressed are just, and the language employed, although sometimes a little turgid, is the language of generous feeling and honest purpose. At present we can spare room for only one or two brief extracts.

"But what constitutes professional character, and what are the best methods of its formation?

In the first place, it is constituted of attainments, intellectual and moral. The mind must be cultivated, and the moral feelings disciplined in order to attain a sound and stable professional character. The intellect must be furnished with a correct and enlarged conception of the subjects pertaining to the science of medicine. No deficiency, derogating from the great beneficent uses of medical knowledge, should exist in the mind of the physician who aspires to the acquisition of an authentic professional character.

The elementary branches of medicine must be thoroughly studied, and after the mind is deeply embued with substantial information on anatomy, physiology, chemistry, general pathology, general therapeutics and materia medica, then the practical branches are to be mastered. Hospital practice in medicine and surgery, as largely contributing to the establishment of a correct judgment of disease, and of the different means of cure, should be assiduously prosecuted. However valuable are the general attainments of the student, and however diversified and minute may be his knowledge of medicine in its principles, yet without personal observation, the recognition and treatment of the various maladies to which mankind are incident, must remain imperfect, and uncertain."

The correctness of these observations must be admitted by every intelligent physician. The remark in reference to Hospital practice is especially important, particularly at a time when the institution of numerous Medical Colleges in places which afford no clinical advantages, makes it the interest of so many to decry the value of such instruction. To what does Philadelphia owe her supremacy as the great seat of medical education in the western world? Undoubtedly to the number, extent, and admirable arrangement of her public institutions, at which clinical advice and instruction are regularly given by her ablest and most experienced physicians and surgeons. At this moment nearly *nine hundred students* are in attendance upon the lectures at her Colleges—a number unexampled in her past history, and unequalled at the present time, except in two or three of the large cities of Europe.

